# Information maximization-based clustering of histopathology images using deep learning

**DOI:** 10.1371/journal.pdig.0000391

**Published:** 2023-12-08

**Authors:** Mahfujul Islam Rumman, Naoaki Ono, Kenoki Ohuchida, MD. Altaf-Ul-Amin, Ming Huang, Shigehiko Kanaya

**Affiliations:** 1 Computational Systems Biology, Nara Institute of Science and Technology, Ikoma, Nara, Japan; 2 Data Science Center, Nara Institute of Science and Technology, Ikoma, Nara, Japan; 3 Department of Surgery and Oncology, Kyushu University, Fukuoka, Kyushu, Japan; Tsinghua University, CHINA

## Abstract

Pancreatic cancer is one of the most adverse diseases and it is very difficult to treat because the cancer cells formed in the pancreas intertwine themselves with nearby blood vessels and connective tissue. Hence, the surgical procedure of treatment becomes complicated and it does not always lead to a cure. Histopathological diagnosis is the usual approach for cancer diagnosis. However, the pancreas remains so deep inside the body that experts sometimes struggle to detect cancer in it. Computer-aided diagnosis can come to the aid of pathologists in this scenario. It assists experts by supporting their diagnostic decisions. In this research, we carried out a deep learning-based approach to analyze histopathology images. We collected whole-slide images of KPC mice to implement this work. The pancreatic abnormalities observed in KPC mice develop similar histological features to human beings. We created random patches from whole-slide images. Then, a convolutional autoencoder framework was used to embed these patches into an integrated latent space. We applied ‘information maximization’, a deep learning clustering technique to cluster the identical patches in an unsupervised manner since our dataset does not have annotation. Moreover, Uniform manifold approximation and projection, a nonlinear dimension reduction technique was utilized to visualize the embedded patches in a 2-dimensional space. Finally, we calculated a few internal cluster validation metrics to determine the optimal cluster set. Our work concentrated on patch-based anomaly detection in the whole slide histopathology images of KPC mice.

## Author summary

Interdisciplinary research is quite advantageous in addressing real-world problems as it brings multiple disciplines into action. A multi-disciplinary approach allows researchers from different backgrounds to work together. It helps increase productivity and understand new ideas. The last decade has witnessed rapid development in digital pathology and opened the path for dealing with pathology differently. As a result, pathological diagnosis is now being explored through the ever-expanding field of artificial intelligence. Deep learning, a subset of artificial intelligence is extremely effective in performing a pixel-based distinction among histopathological structures and thereby, assisting pathologists in their daily practice. In this interdisciplinary research, we analyzed medical data utilizing deep learning algorithms and some statistical tools as well. Statistics helped us in interpreting experimental results. This type of interdisciplinary research will pave the way for better diagnosis of health diseases and in turn, reduce the time and cost of treatment. We verified our experimental results with the help of a pathologist and it was confirmed that our work has significance.

## Introduction

### Research background

Histopathology deals with the examination of microscopic slides incorporating tissues, cells, etc. for the diagnosis of various forms of abnormalities. Histopathologists are expert medical practitioners who inspect cells or tissues under the microscope and make a diagnosis. This is usually the benchmark procedure of histopathological diagnosis. However, it is also a very laborious and tiresome task since these physicists carry out the diagnosis of a huge number of samples every day. The complexity involved in histopathological tissue diagnosis often increases the workload of pathology specialists and it can lead to the reduction of both accuracy and efficiency [1].

Pancreatic cancer (PC) is a deadly disease and it is categorized as one of the most invasive malignancies [2]. According to the data provided by GLOBOCAN 2018, 458918 new cases of PC were registered worldwide which alone contributed to 2.5% of all new cancer diagnoses in that year [3]. PC is a disease in which cancer cells are found in the tissues of the pancreas. Majority of the pancreas-related cancers are of exocrine types that begin in the cells that line the ducts. Pancreatic neuroendocrine tumors are less common compared to exocrine pancreas cancer; however, once eventuated, the smooth function of the healthy cells of the pancreas starts hindering, and it can further result in unimpeded cell division [4]. Pancreatic ductal adenocarcinoma (PDAC) is the 7th leading cause of global cancer which is also responsible for over 90% of pancreatic defects [5].

Despite being one of the least common types of cancer, the survival outcomes of PDAC are extremely low. The reason is that people affected with PC show almost nothing to very few symptoms during the early stages. PDAC has a very dismal prophecy as the survival rate is only 24% after diagnosis, while only 9% live for 5 years [6]. The mortality of pancreas cancer increases with age and is marginally more common in men than women [7]. It is also estimated that 355317 new cases will occur by 2040 all over the globe [7]. It is further approximated that PC will escalate by 18.6 per 100000 people in 2050 around the world [8]. Inter and intra-tumor heterogeneity within cancer cells ascertain the fact that we need satisfactory tools to discover pathological tissue structures as preferred [9].

### Computer-aided diagnosis

Computer-aided diagnosis (CAD) is a system that assists doctors in the explication of medical images. Medical professionals are responsible for analyzing a significant amount of information retrieved from various diagnosis technologies (e.g., X-ray, MRI, CT). CAD systems process digital images for distinctive appearances and focus on prominent features (such as the location of disease) to support a decision taken by expert pathologists. CAD systems help pathologists enhance their diagnostic interpretation by reducing inter-pathologist variations during the diagnostic process [10]. The main goals of CAD include lessening the misdiagnosis rates, saving time and cost concerning medical examinations and reducing the rate of biopsies [11].

In the earlier days, modern computer researchers in various fields explored the possibility of the first CAD systems that utilized flow charts, statistical pattern-matching, probability theory, or knowledge based decision-making processes [12]. However, the attention shifted to data mining approaches later due to various algorithmic restrictions. In recent times, CAD based on deep learning has shown promise in classifying histological structures with high accuracy as artificial intelligence (AI) algorithms can predict malignant growths that can further persuade the therapy process [13]. Medical imaging for diagnosis involving AI is a rapidly growing area of research because of the multidisciplinary nature of machine learning algorithms. CAD is a technology that includes multiple elements like AI, computer vision and medical image processing [14].

Digital medical imaging such as computed tomography (CT), magnetic resonance imaging (MRI) and endoscopic ultrasound can play a vital role in cancer diagnosis and treatment planning [15]. The development and implementation of CAD entail the application of computer technology in medical image elucidation [16]. CAD analysis provides a helping hand to radiologists in characterizing lesions [16]. Computer technology can discover abnormalities in medical images of various modalities that can guarantee early diagnosis to a greater strength [17]. With CAD, radiologists use the computer output as a ‘second opinion’ and it is established on taking the contributions of physicians and computers equally into account [17].

### Deep learning in medical imaging

Deep learning is a subset of machine learning (which is a subset of AI) that uses vast volumes of data and complex algorithms to train a model. Deep learning utilizes neural networks for analyzing data and predicting outcomes. It has found its application in almost every sector such as healthcare, recommendation systems, news customization, image manipulation, music generation, robotics, natural language processing (NLP), object detection, self-driving cars, and so on.

Deep learning has enormous applications in the healthcare sector. It is widely used for drug discovery, cancer detection, and medical research. Deep learning may be used for the automation of various time consuming tasks performed by radiologists such as lesion detection, segmentation, and classification. Appropriate imaging-based classification of numerous diseases is one of the key tasks for radiologists. Deep neural networks (DNNs), especially convolutional neural networks (CNNs) have shown incredible performance in recent years in diagnosing this type of malignancy [18]. CNN is a deep learning algorithm that can process digital images in the form of grid patterns. In [19], CNN has been successfully used for detecting bacterial and viral pneumonia from X-ray images. Another important exercise in medical imaging is biomedical image segmentation. U-Net is a structure that can perform image segmentation [20]. It is one type of convolutional neural network as its architecture can be broadly thought of as an encoder network followed by a decoder network. Mask R-CNN is another neural network framework and state-of-the-art architecture. This variant of DNN can detect objects in a brain MRI image and generate a high quality segmentation mask for each instance. Mask R-CNN extends faster R-CNN by adding a branch for predicting segmentation masks on each ROI (region of interest) [21]. Many problems in computer vision and image processing involve “translating” an input image into a corresponding output image [22]. This task is known as image-to-image translation, where the goal is to learn the mapping between an input image and an output image. Image modality translation in magnetic resonance images has been performed by leveraging a deep learning-based conditional generative adversarial network (cGAN) [23].

Finally, we want to explain the significance of deep learning in the study of histopathology images. With the advent of advanced equipment such as specialized scanning machines, it has become quite easy to store histopathology microscopic glass slides as digital slides on a computer for processing them. Deep learning has become the methodology of choice when it comes to digital histopathology. Deep learning based algorithms including CNNs are distinct from traditional machine learning procedures because of their ability to learn complex representations to perform pattern recognition from raw data without any intervention from human beings [24]. Due to this ability to learn complex representations, the examination of different specimens can be performed accurately by leveraging CNN models.

Morphological variation in histopathology images not only refers to the microscopic appearance of the cancer cell population but also pertains to the growth pattern and stroma of the tumor [25]. For this reason, CNNs are considered superior to traditional multilayer perceptron because of having translational equivariance and translational invariance properties, which can handle inter-class consistency and intra-class variability of complex structured pathological images [26]. The deep learning methods proposed so far in the literature are traditionally based on CNNs, recurrent neural networks (RNNs), generative adversarial networks (GANs), and autoencoders (AEs) [27]. These models refer to different learning strategies such as supervised, weakly supervised, unsupervised, transfer learning and so on. These types of learning strategies are important in the healthcare sector for anomaly detection, disease identification, or tumor segmentation. In our work, we focused on “unsupervised learning”. Unsupervised learning aims at identifying patterns without mapping an input into a predefined output (i.e., label) [27]. Two very popular and heavily used unsupervised learning frameworks are AE and GAN with their multiple variants. In [28], a unified GAN architecture has been implemented that carries out cell-level nuclei segmentation in an unsupervised manner utilizing the haematoxylin and eosin-stained bone marrow histopathology images. In [29], the authors leveraged pre-trained DeepLab V3+ architecture for extracting feature vectors and later applied an unsupervised clustering algorithm on those feature vectors for determining clinically relevant known and unknown features of the kidney at the patient level. Self-supervised learning (SSL) has been used in [30] to perform histopathological image analysis. SSL makes use of the structure within data to auto-generate the labels and it is different from unsupervised learning.

### Objectives of this research

In this research, we leveraged deep learning methodology to distinguish different tissue features of the whole-slide images (WSIs) of KPC mice. A genetically engineered KPC mouse model develops disease progression that corresponds to human beings [31]. A ZEISS Axio Scan.Z1 digital slide scanner (manufactured by Carl Zeiss) was used to digitize the microscopic glass slides. 20× objective lens magnification was applied while converting glass slides into CZI image file format. The pixel resolution under 20× magnification was 0.22 μm per pixel. After that, 30% resizing was exerted to convert CZI files into TIFF format, resulting in a reduction of both image size and resolution. Considering this, the actual resolution will be 0.31 μm per pixel during patch creation from TIFF files. At first, we arbitrarily created small patches from WSIs. We used two different patch sizes: 128×128 and 64×64 pixels. The WSIs were collected from the department of surgery and oncology, graduate school of medical sciences of Kyushu University. We have 191 WSIs in total. Each WSI is stained with 5 distinct staining techniques, namely HE (Haematoxylin & Eosin), MT (Masson’s Trichrome), CD31 (Cluster of Differentiation 31), CK19 (Cytokeratin 19), and Ki67 (Marker of proliferation Ki67) for better visualization of different cell types. The patches have been created in such a way that each of them exhibits the same biological features with different staining. After creating the patches, we fed them to our convolutional autoencoder architecture that embedded these patches into an integrated upper latent space. Convolutional autoencoders are unsupervised deep learning models composed of convolutional layers and they are capable of creating compressed image representations [32]. The compressed representation of the embedded patches is an incorporation of all the staining techniques. Information maximization (IM) was implemented on the lower latent space to perform clustering in the interest of distinguishing different tissue features. IM enlarges the difference between marginal entropy and conditional entropy. It is a soft clustering technique because it assigns a probability to a data point belonging to a particular cluster. In the next section, we will first describe the dataset that will be followed by staining techniques, model architecture, and IM. After that, we will explain the outcome of several internal cluster validation methods. Subsequently, we will describe the clustering results of the optimal cluster set confirmed by internal validation. Finally, Uniform manifold approximation and projection (UMAP) will be shown that visualizes the upper dimensional latent embeddings in a 2-dimensional characterization corresponding to the optimal cluster set.

#### Significance of histological staining

Histological staining is a vital step in diagnosing various diseases as it can provide contrast in tissue sections, rendering the tissue constituents visible for microscopic analysis by medical experts [33]. It is a usual practice in histopathological diagnosis to use some common stains such as haematoxylin and eosin. Histological staining is used to highlight important features of the tissue as well as to differentiate structural elements of the tissue by their color or staining intensity. For example, HE stains cell nuclei, while MT visualizes connective tissue [34]. In immunochemistry, CD31 can be used to demonstrate angiomas and angiosarcomas, which are tumors and cancer cells usually found in the walls of blood vessels or lymphatic vessels. CK19 can detect tumor cells in lymph nodes, peripheral blood and bone marrow of breast cancer patients. Ki67 can be used in immunochemistry and it is strictly associated with cell proliferation.

#### Importance of unsupervised learning

Unsupervised learning can identify patterns in data without annotation and group unsorted information by extracting useful features from them. Unsupervised learning is often focused on clustering as it is the process of grouping objects similar to each other and dissimilar to objects in other clusters. In histopathology study, unsupervised learning, therefore, can discover previously unknown information about tissue by categorizing different cell types in non-identical clusters. Besides, the annotation of medical image data is a very difficult and time-consuming task due to the unavailability of resources and the nature of the process. In this scenario, unsupervised learning turns out to be very useful. Moreover, it is convenient for dealing with quantitative assessment and interobserver variability in pathology.

#### Influence of this research

This research is noteworthy for several reasons. One of them is the automatic separation of different cellular features in the form of patches created from whole-slide images. Our work can be the basis for developing models to handle annotation-free data. This work will show that good clustering can be achieved by considering multiple stainings together. That being the case, information maximization can successfully distinguish between embedded features of multi-stained images and cluster them nicely. It will provide the opportunity to carry out patch-based anomaly detection in the whole-slide images. As a result, the overall condition of the WSI can be observed very easily (e.g., which regions are affected and which are not etc.).

## Materials and method

### Dataset

In this work, we used pathological image patches of KPC mice for conducting our experiment. 191 whole-slide images (WSIs) were collected from the department of surgery and oncology, graduate school of medical sciences of Kyushu University. Each WSI was stained with 5 separate staining techniques. The WSIs were collected as (.tif) files. Each WSI has a dimension of 15000×20000 (Height×Width) pixels. We randomly created small patches from these 191 WSIs and created 15000 samples in total while selecting two different patch sizes: 128×128 and 64×64 pixels. Thus, we created two different datasets, each with 15000 patches but one with 128×128 and the other one with 64×64 pixels spatial size. Hence, the patches in the same positions of these two datasets are not expected to contain the exact biological features because of random creation. However, they correspond to different positions of the WSI that they belong to.

**Fig 1:**
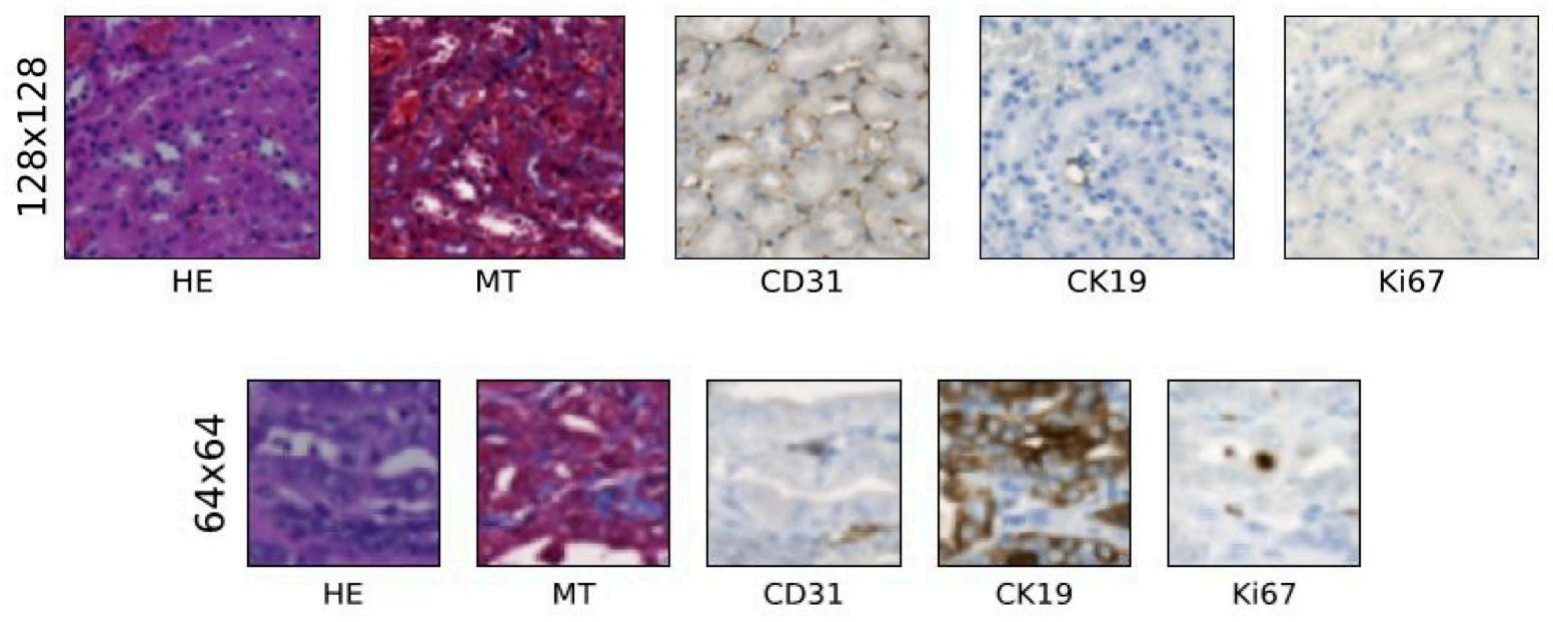
5-stained representation of a patch in a particular position from both datasets. Fig 1 shows a patch in a particular position from both datasets (128×128 and 64×64 pixels).

We can see from Fig 1 that the tissue features in these patches are different due to arbitrary creation from the WSIs.

### Staining techniques

We used 5 different staining techniques for this work: Haematoxylin & Eosin (HE), Masson’s Trichrome (MT), Cluster of Differentiation 31 (CD31), Cytokeratin 19 (CK19), and Marker of proliferation Ki67 (Ki67). HE is used for the demonstration of the nucleus and cytoplasmic inclusions; haematoxylin stains the cell nucleus purple and eosin stains the fibers pink. MT stains muscle and collagen fibers; it highlights the cell nucleus reddish violet as well as collagen fibers blue. CD31 exhibits the presence of endothelial cells in tissue sections. CK19 is a cell marker for cancer stem cells, which are a subpopulation within the tumor. Ki67 is used in oncology to estimate a tumor’s proliferation index.

**Fig 2:**
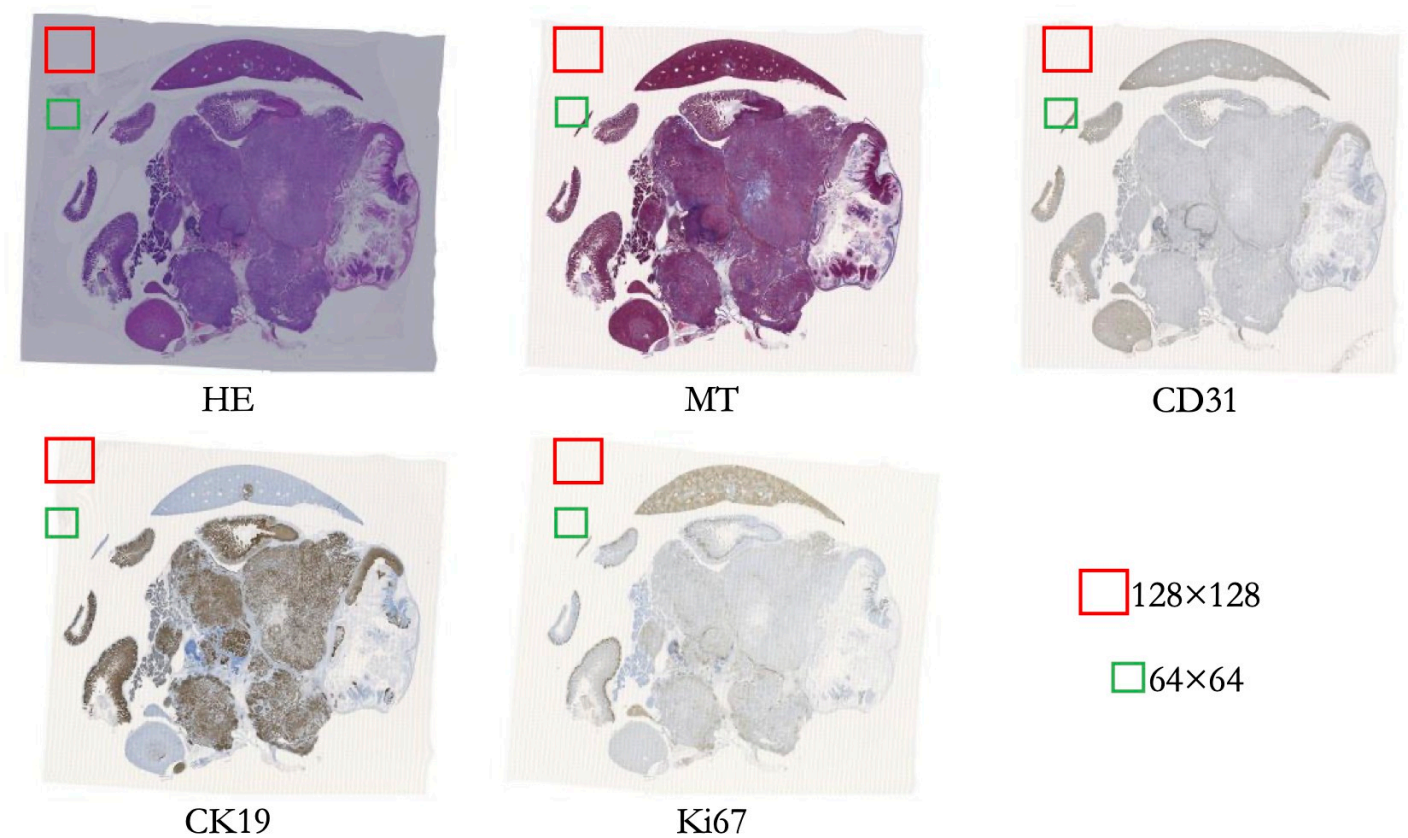
A particular WSI with 5 staining techniques. Fig 2 represents a specified WSI with 5 staining techniques. We have also shown the 2 types of figurative patches (128×128 and 64×64 pixels) that have been created randomly from these WSIs.

The biological features in each WSI shown in Fig 2 are the same although the staining appears to be different to each other. This is true for the patches shown in Fig 1 as well, where each patch represents the same biological features despite looking different to each other.

### Convolutional autoencoder architecture

**Fig 3:**
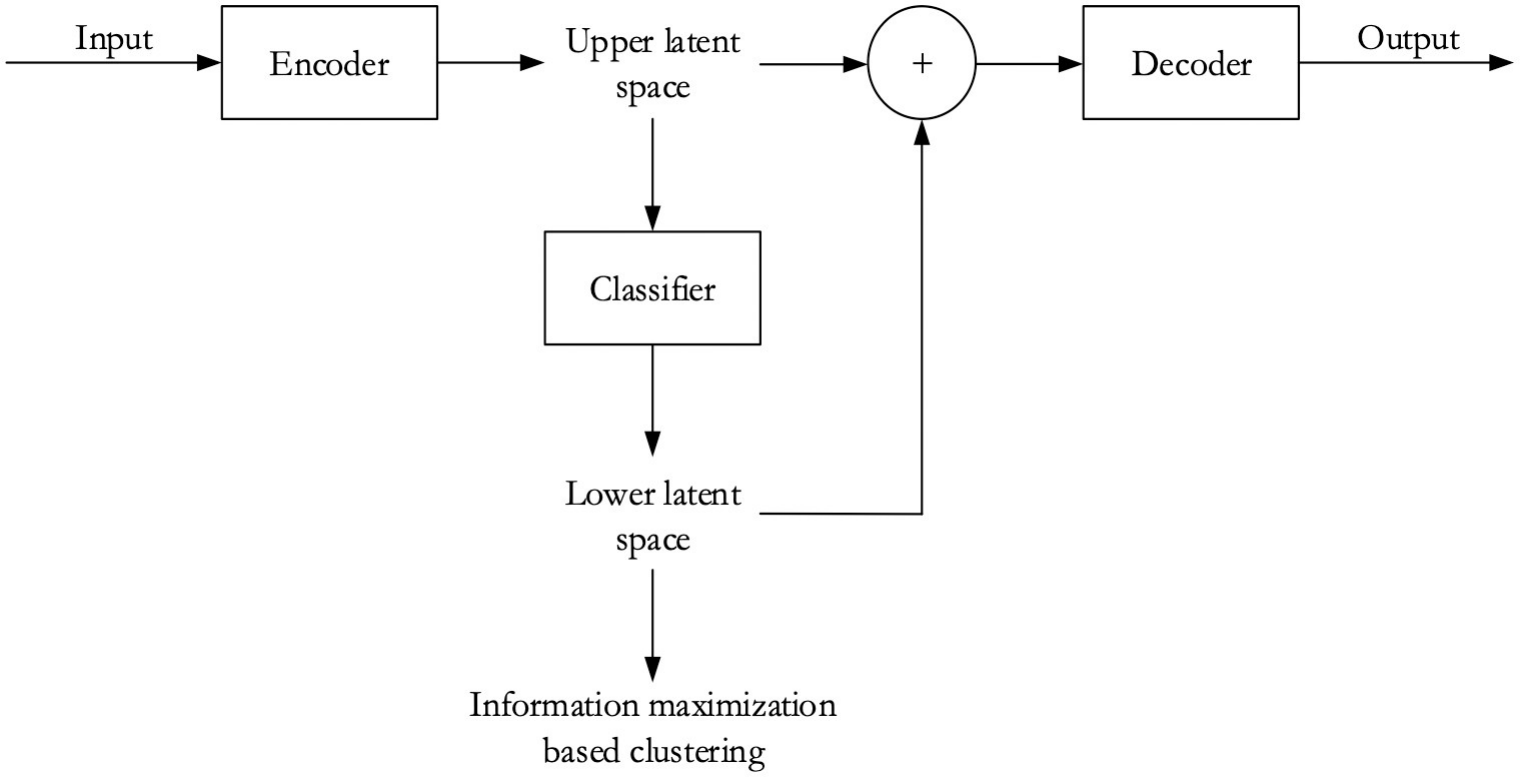
Simplified representation of our convolutional autoencoder architecture. We leveraged a convolutional autoencoder-based deep learning method for our research. Fig 3 shows a simplified representation of our convolutional autoencoder-based deep learning clustering model.

There are essentially three sections in our network: an encoder, a classifier, and a decoder. These sections have been represented with rectangular boxes in Fig 3. Inputs were fed to the encoder portion of our autoencoder architecture. In the case of 128×128 pixels patches, we used rescaled input while training to keep the network depth the same and ensure faster training. We did not do this for 64×64 pixels patches. After the input was processed through the encoder, we obtained upper latent space, which portrays a compressed representation of the input samples. The features in the upper latent space were then passed through the classifier part of the network and reduced further to attain the lower latent space. The output of the classifier, i.e., the feature dimension of lower latent space varies according to the number of clusters of our choosing. Information maximization-based clustering was performed utilizing the lower dimensional latent features. Finally, the features in both the upper latent space and lower latent space were concatenated before being fed to the decoder. The decoder was then tasked with reconstructing the input from it.

### Information maximization

Unsupervised learning is a quite challenging task since it is very much subjective. Finding meaningful patterns from large datasets without annotation is extremely helpful for many applications. Performing unsupervised clustering is equivalent to building a classifier without using annotated samples. In recent times, some researchers have shown improved unsupervised clustering performance by leveraging deep learning. The goal of most of these techniques is to cluster the data points in a way that data with similar characteristics are assigned in the same cluster. Deep learning-based clustering techniques are different from traditional clustering techniques as they cluster the data points by finding complex patterns rather than using simple pre-defined metrics like Euclidean distance or Manhattan distance.

**Fig 4:**
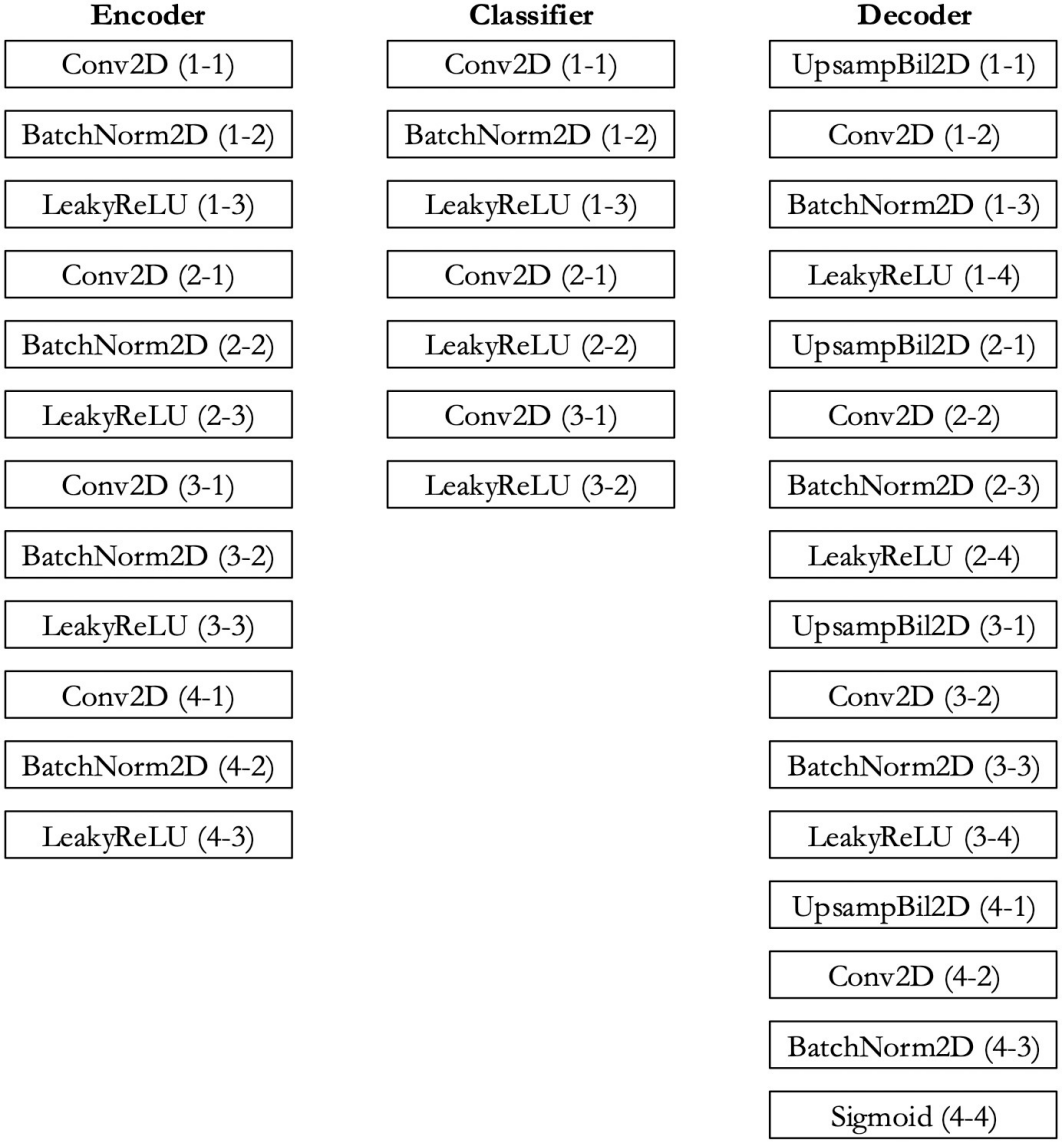
Details of the encoder, classifier and decoder section. Fig 4 presents the encoder, classifier, and decoder sections of our convolutional autoencoder architecture with detailed layer information.

From Fig 4, we can see that there are 12 layers in the encoder, 7 layers in the classifier, and 16 layers in the decoder section. So, our convolutional autoencoder framework is a 35-layer network. The notation ($-#) used in this figure denotes block ($) and layer (#) numbers, respectively. In S1 Table of the “Supporting information”, we have shown how the tensor shape is changed between different sections of our model.

Regularized information maximization is an information theoretic approach to perform clustering which takes care of classifier complexity. This method uses a differentiable loss function with a training objective to maximize the mutual information between the model input and the model output while imposing some regularization penalty on the model parameters. Mutual information can be represented as the difference between marginal entropy and conditional entropy [35]. Hence, the training objective to minimize is

$$\begin{array}{c} R(\theta )-\lambda I(X;Y) \end{array}$$ (1)

Here, R(θ) = regularization penalty; *I*(*X*; *Y*) = mutual information between *X* and *Y*; λ = works as a trade-off. *I*(*X*; *Y*) can be further demonstrated as

$$\begin{array}{c} H(Y)-H(Y|X) \end{array}$$ (2)

where *H*(*Y*) = marginal entropy; *H*(*Y*|*X*) = conditional entropy. So, the purpose of this technique is to maximize the difference between *H*(*Y*) and *H*(*Y*|*X*), i.e., increase the mutual information.

By maximizing *H*(*Y*), diverse cluster assignments are ensured; hence, the model cannot degenerate by assigning all the data points to a single cluster [36]. By minimizing *H*(*Y*|*X*), the cluster assignment of a data point is accomplished with high confidence [36]. Conditional entropy *H*(*Y*|*X*) can be determined by the following equation

$$\begin{array}{c} H\left(Y|X\right)=\frac{1}{N}\sum_{i=1}^{N}h\;(p_{\theta }\left(y|x_{i}\right)) \end{array}$$ (3)

and marginal entropy *H*(*Y*) can be calculated using this equation

$$\begin{array}{c} H\left(Y\right)=h\;(\frac{1}{N}\sum_{i=1}^{N}p_{\theta }\left(y|x_{i}\right)) \end{array}$$ (4)

where *N* denotes the number of samples.

The entropy function for measuring the entropy of each probability distribution can be calculated by using the succeeding equation

$$\begin{array}{c} h(p(y))=-\sum_{y^{\prime }}p\left(y^{\prime }\right)\;\text{log}\;p\left(y^{\prime }\right) \end{array}$$ (5)

The underlying mechanism of the information maximization technique in our work has been described with illustration in S2 Fig of the “Supporting information”. In [37], the DeepInfoMax method has been proposed. In this work, Hjelm et al. maximized the mutual information between the input and output of a deep neural network encoder to learn representations in an unsupervised manner. They did not perform any clustering but focused on training a GAN-like discriminator to differentiate between real and fake samples.

### Loss function

Loss function, which is often regarded as the objective function determines how well a deep learning model performs on the training data. In this research, we defined the loss function *L*_*F*_ as follows

$$\begin{array}{c} L_{F}=R_{L}-(\lambda_{ME}\times H\left(Y\right)-\lambda_{CE}\times H\left(Y|X\right))+\lambda_{AF}\times L_{AF} \end{array}$$ (6)

Here, λ_*ME*_, λ_*CE*_ and λ_*AF*_ are working as trade-offs for getting desirable outcomes for marginal entropy *H*(*Y*), conditional entropy *H*(*Y*|*X*) and affine loss *L*_*AF*_, respectively; *R*_*L*_ here is the reconstruction loss.

#### Reconstruction loss

Autoencoders are unsupervised deep learning models in which we leverage neural networks for the task of representation learning. Specifically, this type of network architecture compresses the knowledge representation of the original input into a bottleneck utilizing a multi-layer encoder. This bottleneck is expected to contain meaningful representations of the given data point. When, this meaningful low-dimensional representation is fed to a decoder, it produces an output of the same size as the input. Hence, this type of network can be trained by minimizing the reconstruction error $L(x,x^{\prime })$, where *x* and *x*′ are the original input and the consequent reconstruction, respectively.

For our work, we used mean squared error (MSE) as the reconstruction loss, which denotes the average of the square of errors [38]. It is computed as the mean of squared differences between the original input and the reconstructed input as shown below

$$\begin{array}{c} \text{MSE}=\frac{1}{n}\;\sum_{i=1}^{n}\;{\left(x_{\text{org},i}-x_{\text{recon},i}\right)}^{2} \end{array}$$ (7)

where *x*_org,*i*_ = original input; *x*_recon,*i*_ = reconstructed input; *n* = number of samples.

#### Affine loss

Affine transformation in deep learning is a transformation that modifies the geometric structure of the image but not the lengths and angles. In this work, we applied rotation, translation, and scaling as affine transformation. The details regarding affine transformation can be found in S3 Fig of the “Supporting information” with a demonstration.

Both the original and the transformed images are provided as input to the encoder section of our convolutional autoencoder architecture. KL (Kullback-Leibler) divergence is an elementary equation that quantifies the closeness of two probability distributions [39]. In our research, KL divergence was measured as the difference between lower dimensional latent features of the original images and transformed images using the following equation

$$\begin{array}{c} D_{KL}\left(P\|Q\right)=\sum_{i}P_{i}\;\text{log}\;(\frac{P_{i}}{Q_{i}}) \end{array}$$ (8)

The deviation between these two distributions is calculated as affine loss and added as a penalty term with the loss function.

### Hyperparameters

Hyperparameters are the variables that determine the network structure of a deep learning model (e.g., number of hidden units) and also the variables that determine how the network is trained (e.g., learning rate) [40].

We listed the hyperparameters used for training the model in Table 1. These hyperparameters include the size of kernels, lengths of strides and so on. We trained our model for 4000 epochs with patch sizes of 128×128 pixels and 3000 epochs with patch sizes of 64×64 pixels. After these many epochs, the increment in mutual information (the difference between the marginal entropy and the conditional entropy) was insignificant. We chose ‘Adadelta’ as the optimizer due to its robustness against noisy gradient information, applicability in different model architectures, effectiveness in various data modalities, and efficacy with the choices of hyperparameters [41].

**Table 1:** List of hyperparameters.

Hyperparameter name	Value
Loss	MSE loss, KL divergence
Hidden layers	15, 45, 128, 196, n_c_
Optimizer	Adadelta
Epochs	4000 (128×128), 3000 (64×64)
Learning rate	0.003
Kernel size	1×1, 3×3, 4×4
Activation function	LeakyReLU, Sigmoid
Batch size	100
Lambda marginal entropy (λ_*ME*_)	0.1
Lambda conditional entropy (λ_*CE*_)	0.03
Lambda affine (λ_*AF*_)	0.03
Stride	1, 2
Padding	0, 1

We have catalogued the list of hyperparameters used in our work in Table 1.

## Results and discussion

### Cluster validation

Cluster validation is used for evaluating the goodness of clustering algorithms. It can be categorized into three classes: internal cluster validation, external cluster validation, and relative cluster validation. In internal validation, only the clustered data is used to evaluate the goodness of clustering without reference to external information. It measures how closely related the objects are in a cluster [42]. In external validation, the result of a cluster analysis is compared to a pre-specified result [43]. Relative validation evaluates the clustering structure by varying parameter values for the same algorithm (e.g., varying the number of clusters in K-Means clustering). Relative criteria can compare two clustering structures and point out the better one in relative term [44].

In this research, we tried to validate our information maximization-based clustering results using 6 cluster validation methods: Xie-Beni index, Calinski-Harabasz index, C index, Hartigan index, Dunn index and Mclain-Rao index. All of these are internal cluster validation techniques and can be applied in our case since our dataset does not have any annotation.

Xie-Beni index focuses on compactness and separation [45]. A smaller Xie-Beni index stipulates a partition in which all the clusters are more compact and separate from each other [46]. Calinski-Harabasz index is a ratio of between-cluster scatter matrix and within-cluster scatter matrix [47]. A higher value of the Calinski-Harabasz index indicates better clustering. The C index is another internal validation index. The sum of all distances between pairs of observations in the same cluster over all clusters is calculated and the sum of the smallest distances between all pairs of points in the entire dataset is subtracted from it; this value is then divided by the subtracted value of the sum of the largest distances between all pairs of points in the entire dataset and the sum of the smallest distances between all pairs of points in the entire dataset [48]. A lower value of the C index denotes better clustering. The Hartigan index is based on the logarithmic relationship between the sum of squares within the cluster and the sum of squares between clusters [49]. The higher the Hartigan index, the higher the clustering quality is. Dunn index is the ratio of the smallest distance between data from different clusters and the largest distance between data in the same cluster [50]. For a given assignment of clusters, a higher Dunn index indicates better clustering (it ranges between 0 to 1). The Mclain-Rao index is defined as the quotient of mean within-cluster and between-cluster distances. A lower value of the Mclain-Rao index is desirable.

From Table 2, we can see that among these 6 metrics, three (Xie-Beni index, Calinski Harabasz index and C index) denoted 14 as the optimal cluster set while the Dunn index selected 13 and two metrics (Hartigan index and Mclain-Rao index) chose 15 as optimal for the case of 128×128. On the other hand, three metrics (Hartigan index, Dunn index and Mclain-Rao index) chose 14 as the best cluster set while the Xie-Beni index, Calinski-Harabasz index and C index selected 13, 11 and 17 as optimal, respectively for the case of 64×64. Therefore, out of these 12 instances demonstrated by the 6 metrics mentioned above, 6 instances have shown 14 as the optimal number of clusters for both patch sizes; 13 and 15 each have been shown by 2 instances; 11 and 17 each have been shown by 1 instance. For this reason, we have chosen 14 as the optimal number of clusters in both cases.

**Table 2:** Internal validation indices for different cluster sets (128×128 and 64×64).

Cluster validation indices	128×128		64×64	
Xie-Beni index	Cluster set	Value	Cluster set	Value
	8	210.56600	8	208.66015
	9	123.37128	9	94.03908
	10	269.10936	10	99.14787
	11	109.27157	11	85.44762
	12	197.86335	12	108.40803
	13	83.87588	13	**76.72227**
	14	**77.13336**	14	91.36067
	15	89.88902	15	164.86141
	16	88.22176	16	113.37091
	17	107.32371	17	740.29343
	18	130.49625	18	182.85043
Calinski-Harabasz index	Cluster set	Value	Cluster set	Value
	8	3417.12372	8	1556.83725
	9	3068.88135	9	2684.95843
	10	1854.72219	10	2934.73878
	11	3268.10641	11	**3297.37070**
	12	2415.86019	12	2784.55430
	13	3405.33049	13	2862.23775
	14	**3458.73942**	14	2692.61013
	15	3335.13698	15	2097.78152
	16	2616.69806	16	2320.94120
	17	2582.27002	17	1267.71892
	18	2536.53023	18	1317.30883
C index	Cluster set	Value	Cluster set	Value
	8	0.11338	8	0.11185
	9	0.11750	9	0.11189
	10	0.10734	10	0.09697
	11	0.10647	11	0.08767
	12	0.11046	12	0.08885
	13	0.09428	13	0.08772
	14	**0.08816**	14	0.08332
	15	0.09366	15	0.10025
	16	0.10316	16	0.07896
	17	0.08943	17	**0.07132**
	18	0.09558	18	0.07615
Hartigan index	Cluster set	Value	Cluster set	Value
	8	0.46719	8	-0.31895
	9	0.49330	9	0.35966
	10	0.10758	10	0.56646
	11	0.77948	11	0.78839
	12	0.57270	12	0.71473
	13	1.00307	13	0.82933
	14	1.09874	14	**0.84834**
	15	**1.13652**	15	0.67289
	16	0.96298	16	0.84304
	17	1.01434	17	0.30289
	18	1.05716	18	0.40195
Dunn index	Cluster set	Value	Cluster set	Value
	8	0.00697	8	0.00734
	9	0.01016	9	0.01075
	10	0.00727	10	0.00873
	11	0.00763	11	0.01132
	12	0.00592	12	0.00957
	13	**0.01360**	13	0.01146
	14	0.01128	14	**0.01307**
	15	0.01060	15	0.00670
	16	0.01104	16	0.01060
	17	0.00704	17	0.00335
	18	0.00981	18	0.00674
Mclain-Rao index	Cluster set	Value	Cluster set	Value
	8	0.49971	8	0.52964
	9	0.49636	9	0.49275
	10	0.51922	10	0.45613
	11	0.45388	11	0.44574
	12	0.47768	12	0.44275
	13	0.42899	13	0.44423
	14	0.43028	14	**0.43151**
	15	**0.42482**	15	0.46676
	16	0.44847	16	0.43344
	17	0.42538	17	0.45157
	18	0.42538	18	0.46498

Table 2 represents 6 internal cluster validation metrics for different cluster sets using 128×128 and 64×64 pixels patches.

**Fig 5:**
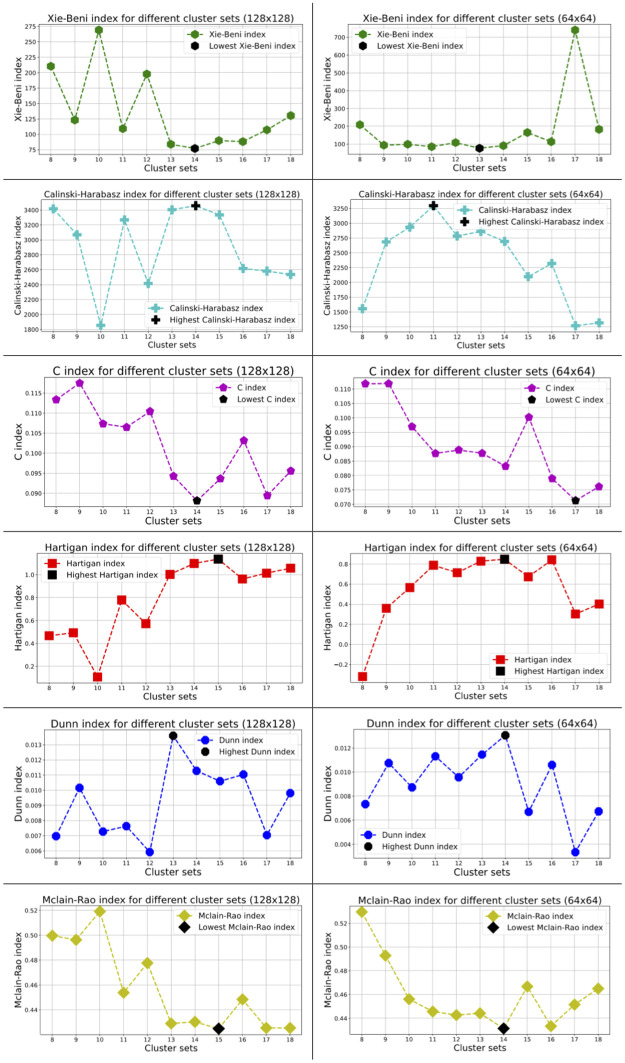
Various internal cluster validation indices for different cluster sets (128×128 and 64×64). Fig 5 shows the values of various internal validation indices for different clusters using both 128×128 and 64×64 pixels patches.

Fig 5 displays the plotting for different internal cluster validation indices for the selected number of clusters using both 128×128 and 64×64 pixels patches. The optimal number of clusters for each validation index can be easily detected from Fig 5.

### Clustering results

Cluster analysis or clustering is the task of grouping a set of objects in such a way that objects in the same group (called a cluster) are more similar (in some sense) to each other than those in other groups (clusters).

A pathology specialist determined the names of the clusters obtained using 128×128 pixels patches. These names are provided below.

Cluster 1: Cancer cells
Cluster 2: Liver, Kidney
Cluster 3: Blank
Cluster 4: Intestinal mucosa, Spleen
Cluster 5: Vascular endothelium
Cluster 6: Tissue gap
Cluster 7: Cancer cells with rich stroma
Cluster 8: Liver peripheral
Cluster 9: Liver center
Cluster 10: Tissue stump
Cluster 11: Cancer cells
Cluster 12: Liver
Cluster 13: Dense small blood vessels
Cluster 14: Intestinal mucosa

**Fig 6:**
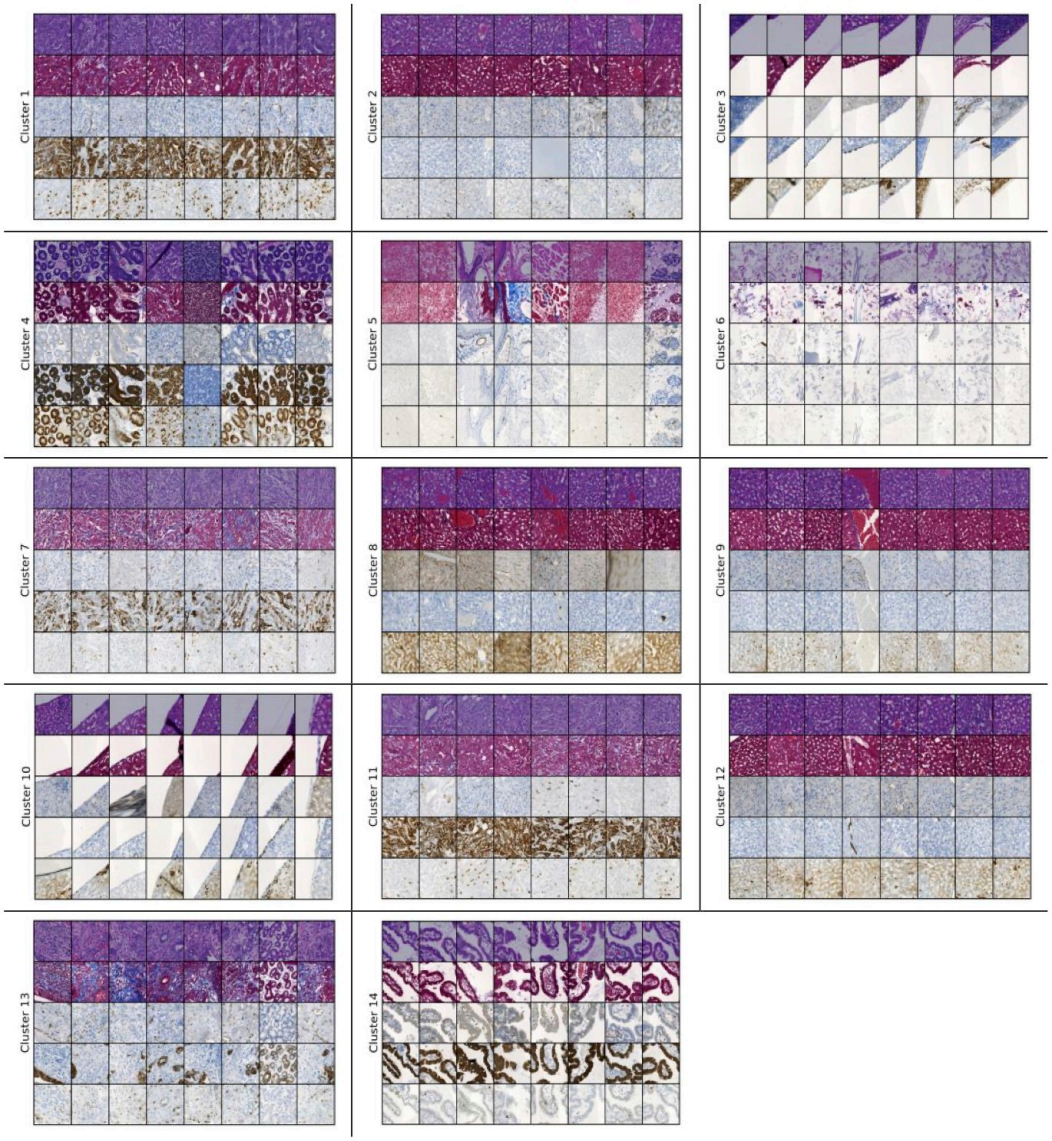
Clustering results of the 14-cluster set (128×128). Fig 6 displays the clustering results of the 14-cluster set obtained using the 15000 samples of size 128×128 pixels.

We would like to mention that the pathologist determined the names of the clusters shown in Fig 6 by examining the colormap (which shows different regions with different colors) of a randomly selected WSI out of the 191 that we had available. We explained more about this in detail later. We can notice some types of cancer cells in three clusters (1, 7 and 11). Pancreatic cancer is well known to be heterogeneous in its molecular and morphological phenotype [51].

**Fig 7:**
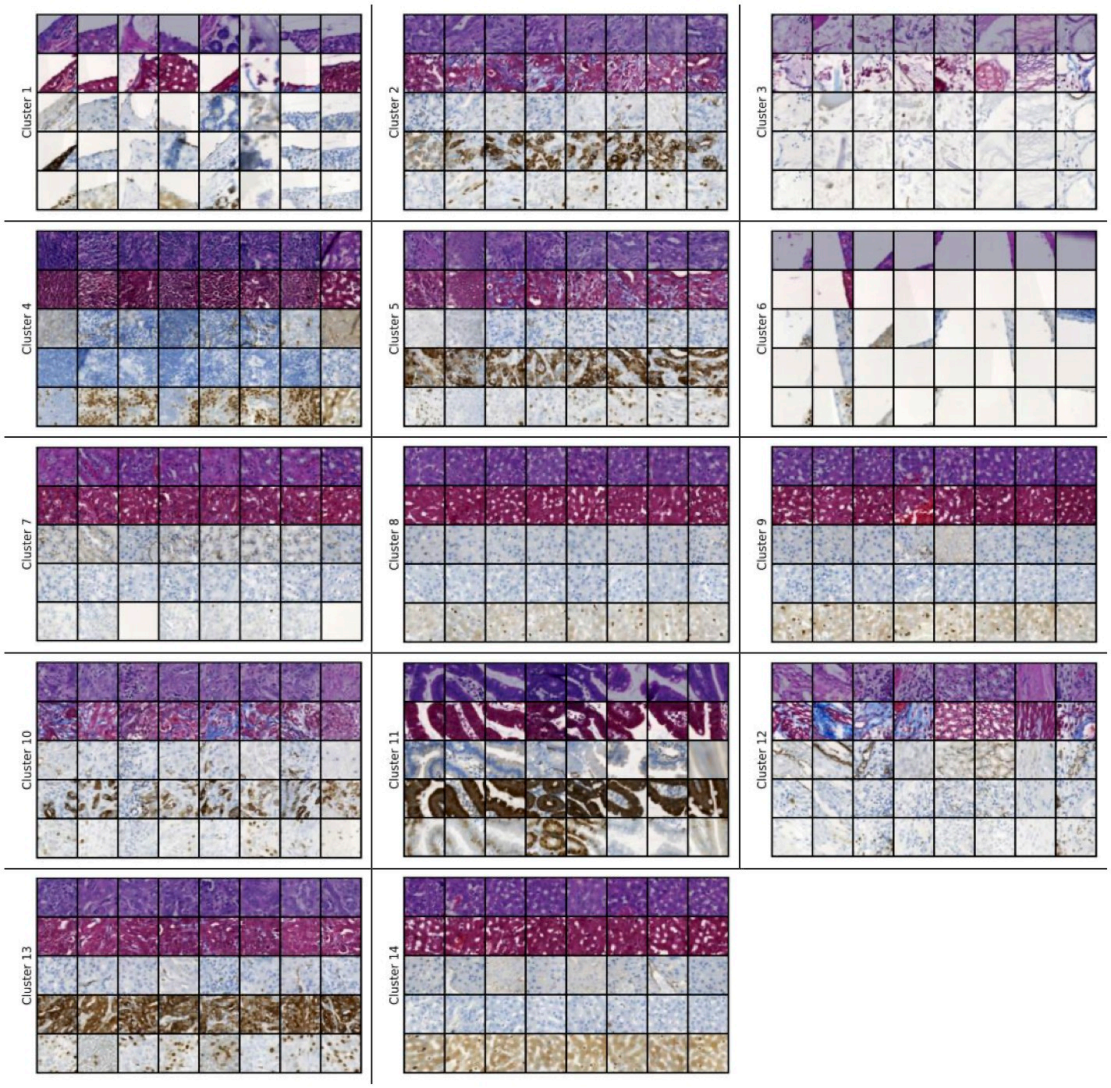
Clustering results of the 14-cluster set (64×64). Fig 7 displays the clustering results of the 14-cluster set obtained using the 15000 samples of size 64×64 pixels.

Now, we will present the clustering results of the 14-cluster set attained with the 64×64 pixels patches. The number of samples is 15000 in this case as well which were created from 191 WSIs. The pathologist did not provide annotations for the clusters displayed in Fig 7, but we can notice a lot of similarities with 128×128 patches.

### Embedded features visualization

We carried out this inquisitive unsupervised learning task for cluster sets of 8, 9, 10, 11, 12, 13, 14, 15, 16, 17, and 18 using data with two different patch sizes. These are the values of n_c_. After the completion of training, we obtained embedded features as encoded variables from the upper latent space and a probability distribution for each patch from the lower latent space. We leveraged UMAP for visualizing the embedded features in a 2-dimensional space.

**Fig 8:**
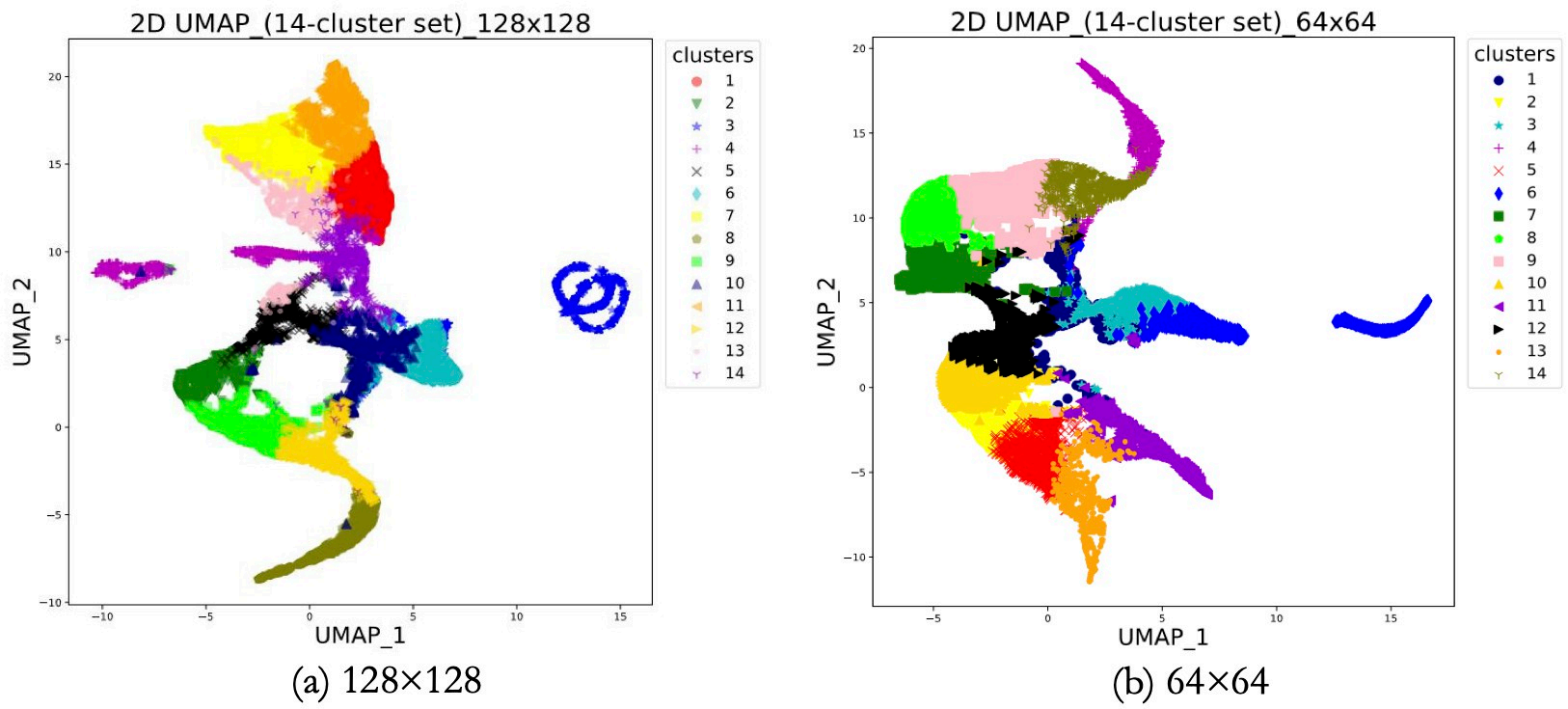
2D UMAP for 14-cluster set using 128×128 and 64×64 pixels patches. Fig 8 exhibits the visualization of the embedded features of the 14-cluster set corresponding to 128×128 (part a) and 64×64 (part b) pixels patches.

From Fig 8, we can notice that there is no clear boundary between the clusters. Nevertheless, the data points at distant places can be assigned to different clusters. UMAP shows that the clusters are not widely separated (adjacent to each other). The encoder mapped the patches in this dataset within very little space and for this reason, when we visualized them in 2-dimensional space, they appeared the way they are seen from the UMAP plotting. UMAP is a novel manifold learning technique for dimension reduction. It has no computational restrictions on the embedding dimension, thus making it feasible for a general-purpose dimension reduction technique [52]. We reduced the dimension of embedded features down to 2 for both datasets.

### Patch-based anomaly detection in WSI using the trained model

**Fig 9:**
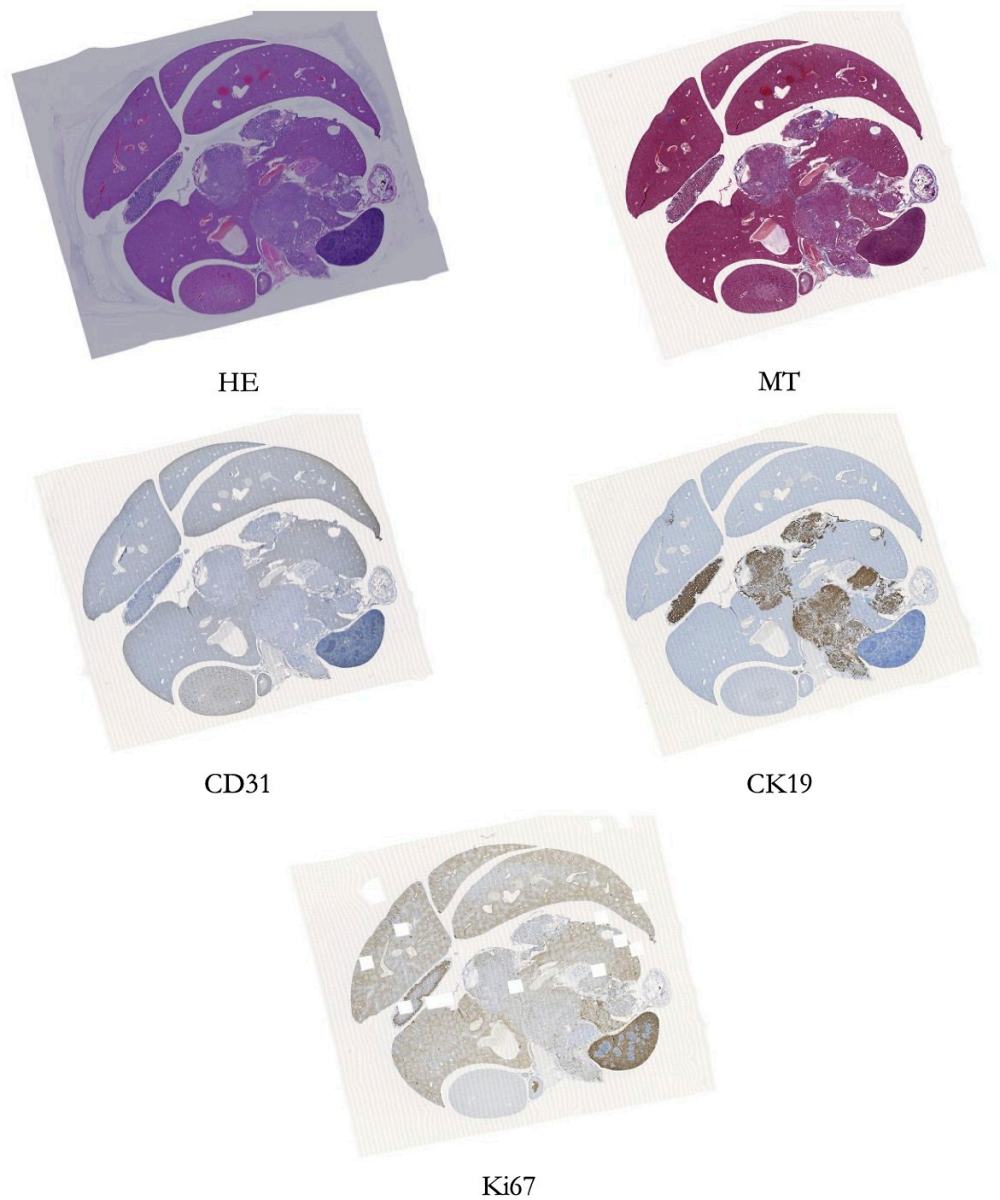
A specified WSI used for sequential patch creation. Fig 9 shows our randomly selected WSI with 5 staining techniques (HE, MT, CD31, CK19 and Ki67). We used it to create the 18252 patches without overlap of size 128×128.

We carried out a patch-based anomaly detection by choosing one WSI randomly out of 191. Fig 9 reveals this specified WSI. We already mentioned that the size of each WSI is 15000×20000 (Height×Width) pixels. We created 128×128 pixels patches sequentially (without overlap) from this WSI which provided us with 18252 patches (from one WSI). After that, these patches were fed to the 14-cluster model for 128×128 pixels patches. Subsequently, we obtained cluster IDs for each of those patches and we used them to create a colormap of this particular WSI.

**Fig 10:**
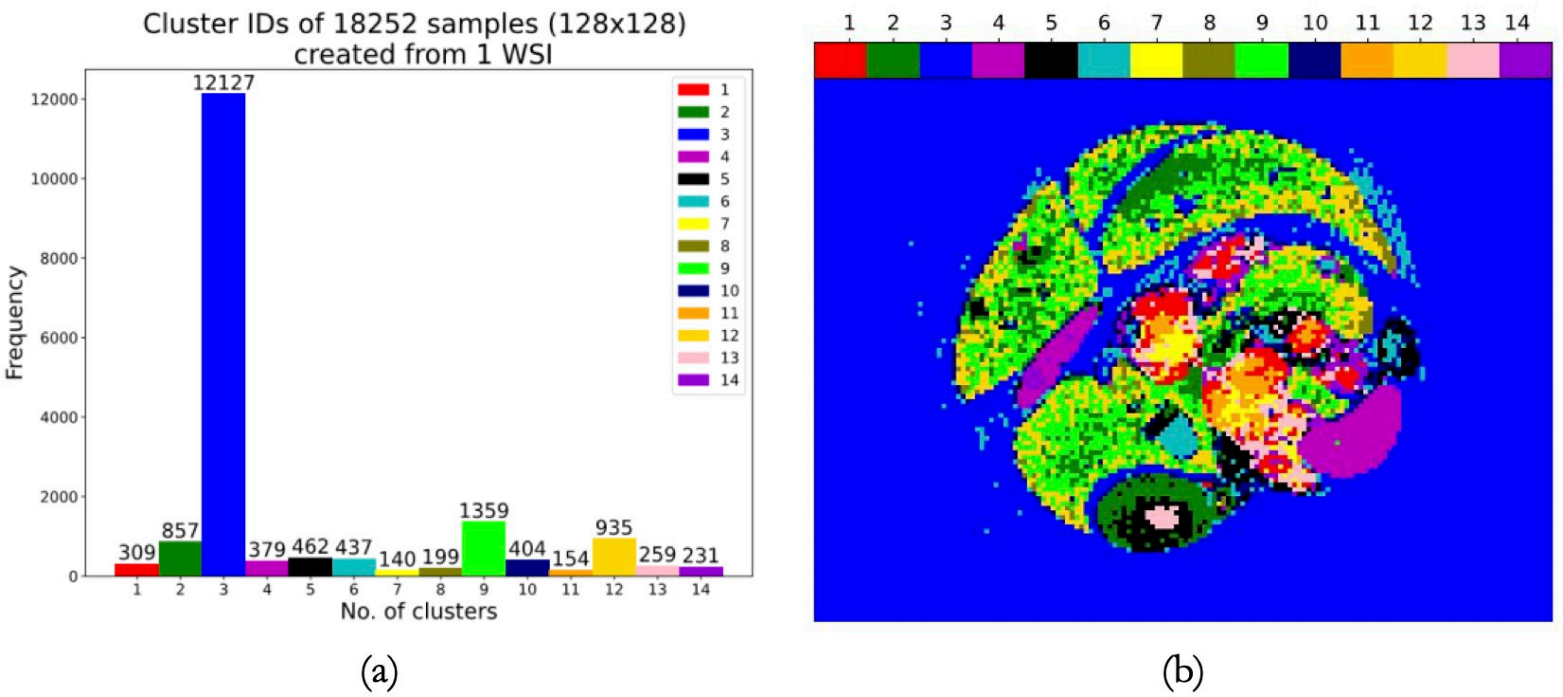
A histogram showing cluster IDs and a colormap representing them with designated colors. Fig 10 shows a colormap (part b) where different cluster IDs of 18252 patches have been indicated with designated colors. A histogram (part a) has also been shown which represents the frequency of the samples (among 18252 patches) in each cluster. The same procedure can be done using 64×64 pixels patches as well but here, we have only done it using 128×128 patches. We are repeating our earlier statement that the pathologist used the multi-stained representation of this particular WSI and its colormap to name various tissue features of the 14-cluster set using patches of 128×128 pixels.

From the histogram (part a) of Fig 10, we observe that 309 patches have been categorized as cancer cells (cluster 1); 140 are termed cancer cells with rich stroma (cluster 7). 154 samples (cancer cells) have gone to cluster 11. Clusters 2, 8, 9 and 12 have 857, 199, 1359 and 935 patches, respectively. Cluster 2 contains the liver and kidney; cluster 8 has the liver’s peripheral tissue; cluster 9 includes the liver center and cluster 12 holds liver tissue. Cluster 3 (blank) accumulated 12127 patches; cluster 6 (tissue gap) assembled 437 patches; cluster 10 (tissue stump) gathered 404 patches. 379 samples in cluster 4 denote intestinal mucosa and spleen. 462 samples in cluster 5 indicate vascular endothelium. 259 patches in cluster 13 portray dense small blood vessels. 231 specimens depict intestinal mucosa in cluster 14. We can see the positioning of these different patches in the colormap (part b) of our arbitrarily chosen WSI. In this way, we can understand a WSI’s overall condition from our experiment. The pathologist also confirmed that the pancreas region of this particular WSI has been heavily replaced by pancreatic cancer and there is no normal cell available in the pancreas of this WSI. In other terms, cancer cells in this WSI originated from the pancreas.

## Conclusion

In this study, we mainly performed pathological image analysis using whole-slide histopathology images of KPC mice. We leveraged deep learning methodology based on convolutional autoencoder architecture to categorize different tissue features of the pancreas in distinct clusters. At first, we randomly created small patches from whole-slide images (WSIs) which were prepared with 5 different staining techniques. We embedded these patches into an integrated latent space using our deep learning model. We utilized information maximization, an unsupervised clustering technique that accomplishes the task of separating different histological features into distinguishable clusters. We also visualized the clustering structure of the embedded features by employing UMAP in a 2-dimensional space. Additionally, we confirmed the optimal number of clusters using several internal cluster validation metrics. We also carried out patch-based anomaly detection in a WSI and represented different patches with specific colors in a colormap.

We used two different patch sizes for conducting this experiment: 128×128 and 64×64 pixels. We obtained the 14-cluster set as the optimal number of clusters for both patch sizes. We obtained the names of different types of tissue features for 128×128 pixels patches after consulting with a pathologist. They have recognizable properties on their own. The optimal number of clusters obtained by internal validation indices indicates data in these particular cluster sets (14 in both cases) are more similar to each other than other cluster sets.

Deep learning algorithms are referred as “black boxes” and it’s often unclear why a particular algorithm works. Hence, a way is required for AI to present information to experts on which to base its predictions, and for expert personnel to verify the outputs of the AI [53]. Using such human-AI interfaces, medical practitioners will be able to make more credible decisions with a high level of understanding.

The significance of this research is that it can carry out patch-based anomaly detection in whole-slide images. It can assist professionals by detecting cancer in the WSIs. Besides, computer-aided diagnosis (CAD) plays an important role in helping medical practitioners make diagnostic decisions.

- The code for this work can be found at this address: https://github.com/randomaccess2023/KPC_IM_128_64.
- The datasets (128×128 and 64×64 pixels) can be located here: https://doi.org/10.6084/m9.figshare.24129360.
- The dataset obtained from sequential patch creation can be accessed from this link: https://doi.org/10.6084/m9.figshare.24137553.

### Limitations

We already mentioned that we randomly created 15000 samples of two different patch sizes (128×128 and 64×64 pixels) from 191 WSIs. After analyzing the clustering results, we acquired 14-cluster set as optimal (using internal validation indices) in the case of both 128×128 and 64×64 pixels patches. However, since we created these patches randomly, we cannot guarantee whether our dataset contains all the distinctive tissue features that can be extracted from the WSIs. Furthermore, the patches used in this experiment do not have annotation and therefore, external cluster validation is not possible in this research.

## Supporting information

S1 Table Summary of encoder, classifier and decoder section. (PDF)

S1 Fig Information maximization technique. (PDF)

S2 Fig Original vs. transformed image (128×128). (PDF)

S3 Fig The learning curve (64×64). (PDF)

S4 Fig Information maximization output for the 14-cluster set (64×64). (PDF)

S5 Fig Information maximization output of unused samples (128×128) during training using the 14-cluster set model. (PDF)
