# Supplementary material for: Information maximization-based clustering of histopathology images using deep learning
Source: PLOS Digit Health. 2023 Dec 8;2(12):e0000391. doi: 10.1371/journal.pdig.0000391 (PMC10707605; doi:10.1371/journal.pdig.0000391)
Supplement: S1 Table — (PDF) [file pdig.0000391.s001.pdf]

Supporting information: S1 Table

Change of tensor shape within convolutional autoencoder architecture

We mentioned in the 'Convolutional autoencoder architecture' subsection of the "Materials and method" section of our manuscript that there are 12 layers in the encoder, 7 layers in the classifier, and 16 layers in the decoder section. Hence, our convolutional autoencoder framework is a 35-layer network. We have shown how the tensor shape is changed in the S1 Table.

S1 Table. Summary of encoder, classifier and decoder section.

Encoder layers	Output shape (B, C, H, W)
Conv2D-1	[-1, 45, 32, 32]
BatchNorm2D-2	[-1, 45, 32, 32]
LeakyReLU-3	[-1, 128, 32, 32]
Conv2D-4	[-1, 128, 16, 16]
BatchNorm2D-5	[-1, 128, 16, 16]
LeakyReLU-6	[-1, 128, 16, 16]
Conv2D-7	[-1, 196, 8, 8]
BatchNorm2D-8	[-1, 196, 8, 8]
LeakyReLU-9	[-1, 196, 8, 8]
Conv2D-10	[-1, 128, 4, 4]
BatchNorm2D-8	[-1, 128, 4, 4]
LeakyReLU-12	[-1, 128, 4, 4]

Classifier layers	Output shape (B, C, H, W)
Conv2D-1	[-1, 128, 1, 1]
BatchNorm2D-2	[-1, 128, 1, 1]
LeakyReLU-3	[-1, 128, 1, 1]
Conv2D-4	[-1, 128, 1, 1]
LeakyReLU-5	[-1, 128, 1, 1]
Conv2D-6	[-1, n_c, 1, 1]
LeakyReLU-7	[-1, n_c, 1, 1]

Decoder layers	Output shape (B, C, H, W)
UpsampBil2D-1	[-1, 128+n_c, 8, 8]
Conv2D-2	[-1, 196, 8, 8]
BatchNorm2D-3	[-1, 196, 8, 8]
LeakyReLU-4	[-1, 196, 8, 8]
UpsampBil2D-5	[-1, 196, 16, 16]
Conv2D-6	[-1, 128, 16, 16]
BatchNorm2D-7	[-1, 128, 16, 16]
LeakyReLU-8	[-1, 128, 16, 16]
UpsampBil2D-9	[-1, 128, 32, 32]
Conv2D-10	[-1, 45, 32, 32]
BatchNorm2D-11	[-1, 45, 32, 32]
LeakyReLU-12	[-1, 45, 32, 32]
UpsampBil2D-13	[-1, 45, 64, 64]
Conv2D-14	[-1, 15, 64, 64]
BatchNorm2D-15	[-1, 15, 64, 64]
Sigmoid-16	[-1, 15, 64, 64]

In the S1 Table, (B, C, H, W) in the output shape stands for `batch_size`, `channels`, `height` and `width`, respectively. The value of `batch_size` -1 indicates that this dimension is variable and depends on our choice. In the classifier section of this table, n_c will vary according to the number of clusters that we select. We can also see that the final output shape from the decoder is [-1, 15, 64, 64], where 15 is the number of `channels` and 64×64 is the spatial size of the output. In autoencoder architectures, the input shape and the output shape of the network are the same. So, the input that we fed to the encoder had the shape [-1, 15, 64, 64] too. As we already stated, we used 5 staining techniques (HE, MT, CD31, CK19 and Ki67) to represent each patch in this work. As a patch with distinct staining has 3 color channels in it; when we concatenated the 5 stained representations of each patch on the channel axis, the number of channels became 15.
