# Supplementary material for: Information maximization-based clustering of histopathology images using deep learning
Source: PLOS Digit Health. 2023 Dec 8;2(12):e0000391. doi: 10.1371/journal.pdig.0000391 (PMC10707605; doi:10.1371/journal.pdig.0000391)
Supplement: S1 Fig — (PDF) [file pdig.0000391.s002.pdf]

Supporting information: S1 Fig

The underlying mechanism of the information maximization technique

In the 'Information maximization' subsection of the "Materials and method" section of the paper, we talked about information maximization from the mathematical point of view. Here, we will explicate the underlying mechanism of this technique in our research. S1 Fig portrays the operations that take place on the tensors obtained from the classifier section of our convolutional autoencoder architecture explained in Fig 4 of the manuscript and S1 Table elaborately. Here, the rows (denoted by N's) represent all the samples (patches) and the columns (symbolized by C's) depict all the clusters. The P's here denote the probability distribution for each sample.

In one case, the entropy is calculated along each row, i.e., for each sample as shown in green (CE) in S1 Fig and then the average is calculated. In another case, the average of the probability distributions is computed along each column, i.e., for each cluster and then entropy is determined as shown in red (ME) in S1 Fig. Marginal entropy (ME) and conditional entropy (CE) can be calculated using equations (3) and (4), respectively as shown in the manuscript. Maximization of marginal entropy ensures diverse cluster assignments. It will make the distribution of clusters uniform as marginal entropy will be high when dissimilar data points go to different clusters. Minimizing conditional entropy guarantees the cluster assignment of a data point with high confidence.

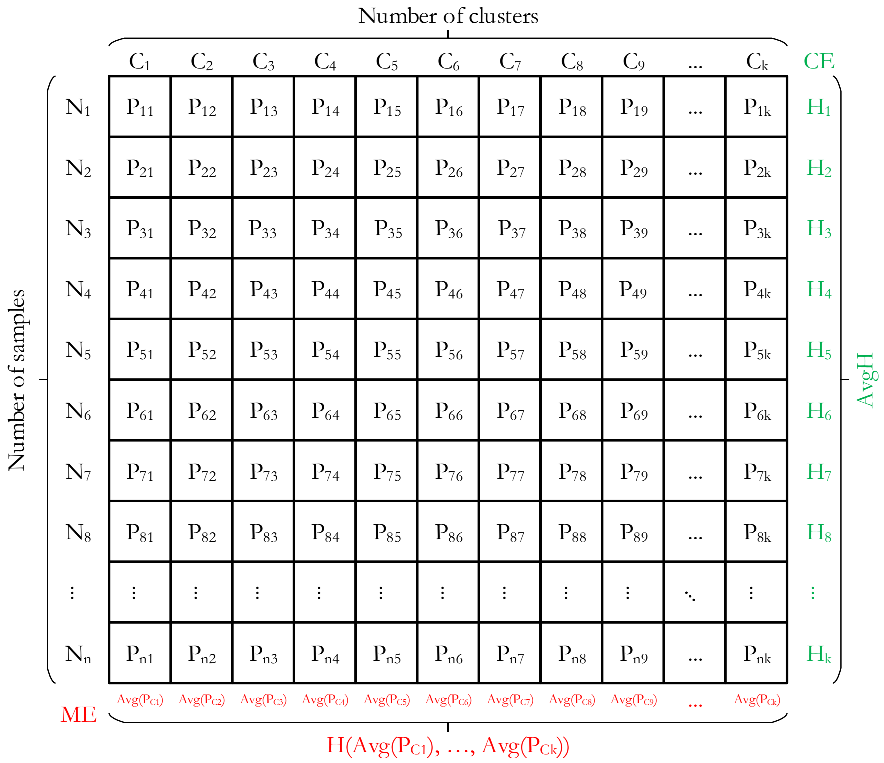

S1 Fig. Information maximization technique.
