# Supplementary material for: Information maximization-based clustering of histopathology images using deep learning
Source: PLOS Digit Health. 2023 Dec 8;2(12):e0000391. doi: 10.1371/journal.pdig.0000391 (PMC10707605; doi:10.1371/journal.pdig.0000391)
Supplement: S2 Fig — (PDF) [file pdig.0000391.s003.pdf]

Supporting information: S2 Fig

Affine transformation

Affine transformation in deep learning is a transformation that modifies the geometric structure of the image but not the lengths and angles. In this work, we applied rotation, translation, and scaling as affine transformation. Rotation rotates each input image randomly within a certain range of degrees. Translation moves an image towards the horizontal and vertical axis in a random manner. Scaling readjusts the spatial dimension of the image. We showed the combined effects of these three techniques in S2 Fig.

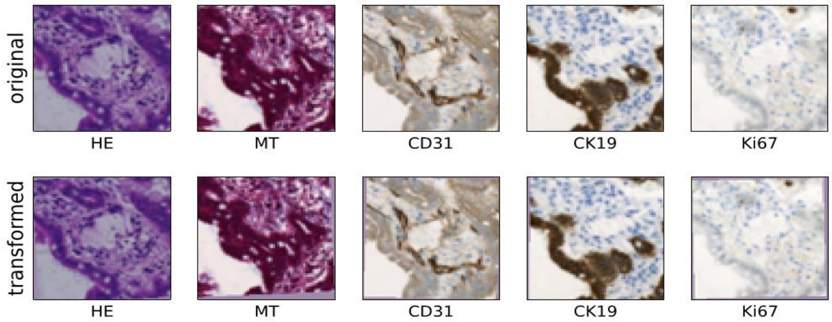

S2 Fig. Original vs. transformed image (128×128).

S2 Fig shows the difference between the original image (before transformation) and the transformed image for 128×128 pixels patches using 5 staining techniques. We did not show this effect for 64×64 pixels patches to avoid repetitiveness. We can witness that some blank spaces occur around the borders of the transformed images. We used pixel fill values for the area outside the transformed image using the average pixel values of RGB channels in our dataset.
