# Supplementary material for: Information maximization-based clustering of histopathology images using deep learning
Source: PLOS Digit Health. 2023 Dec 8;2(12):e0000391. doi: 10.1371/journal.pdig.0000391 (PMC10707605; doi:10.1371/journal.pdig.0000391)
Supplement: S3 Fig — (PDF) [file pdig.0000391.s004.pdf]

Supporting information: S3 Fig

The learning curve

After training the model for 4000 and 3000 epochs with 128×128 and 64×64 pixels patches, respectively, we observed that the difference between marginal entropy and conditional entropy (mutual information) was gradually increasing, while the value of the loss function was progressively decreasing. This can be understood from the S3 Fig.

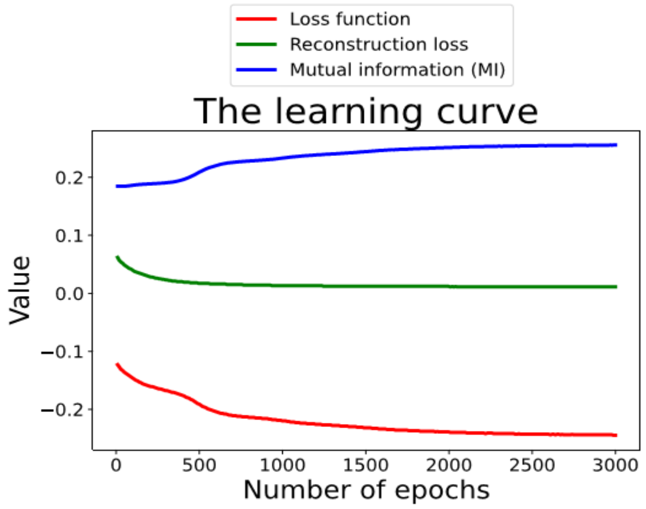

S3 Fig. The learning curve (64×64).

S3 Fig shows the learning curve for the model trained using the 64×64 pixels patches. Here, the three lines represented with red, green, and blue denote loss function, reconstruction loss, and mutual information, correspondingly. The learning curve obtained for the model trained using the 128×128 pixels patches is utterly similar to this one; hence, we decided to show only one.
