# Supplementary material for: Information maximization-based clustering of histopathology images using deep learning
Source: PLOS Digit Health. 2023 Dec 8;2(12):e0000391. doi: 10.1371/journal.pdig.0000391 (PMC10707605; doi:10.1371/journal.pdig.0000391)
Supplement: S4 Fig — (PDF) [file pdig.0000391.s005.pdf]

Supporting information: S4 Fig

Information maximization output

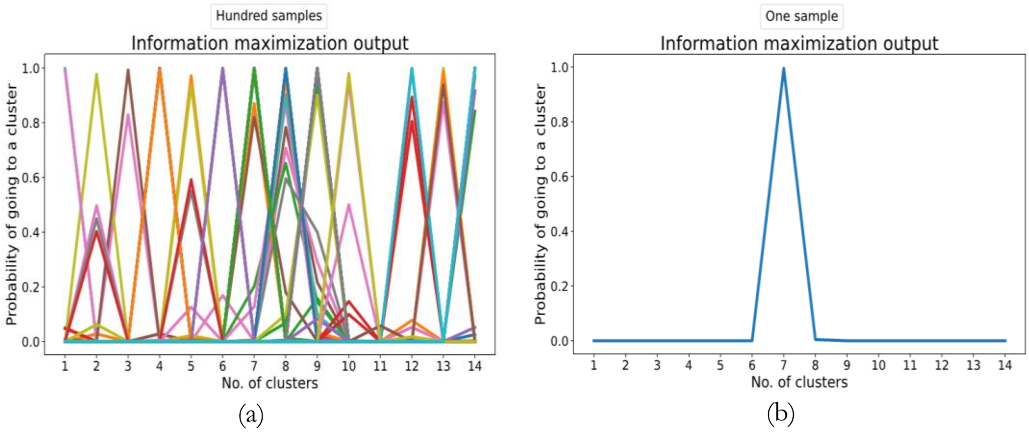

S4 Fig. Information maximization output for the 14-cluster set (64×64).

S4 Fig shows the information maximization output of the 14-cluster set using 64×64 pixels patches. Here, part (a) shows the probability of going to a cluster for 100 arbitrarily chosen samples from our dataset. For better understanding, we have provided another example in part (b). Here, we can see that the probability for this particular sample to be in cluster 7 is the highest. Information maximization can be considered a soft clustering technique where a probability is assigned to a specific sample for going to a certain cluster. In this case in part (b), this sample has the highest probability of belonging in cluster 7 and a very low probability of belonging in the other clusters. Information maximization output for the model trained using the 128×128 pixels patches is quite similar to this one; hence, we showed only one.
