# Supplementary material for: Information maximization-based clustering of histopathology images using deep learning
Source: PLOS Digit Health. 2023 Dec 8;2(12):e0000391. doi: 10.1371/journal.pdig.0000391 (PMC10707605; doi:10.1371/journal.pdig.0000391)
Supplement: S5 Fig — (PDF) [file pdig.0000391.s006.pdf]

Supporting information: S5 Fig

Information maximization output of unused patches during training

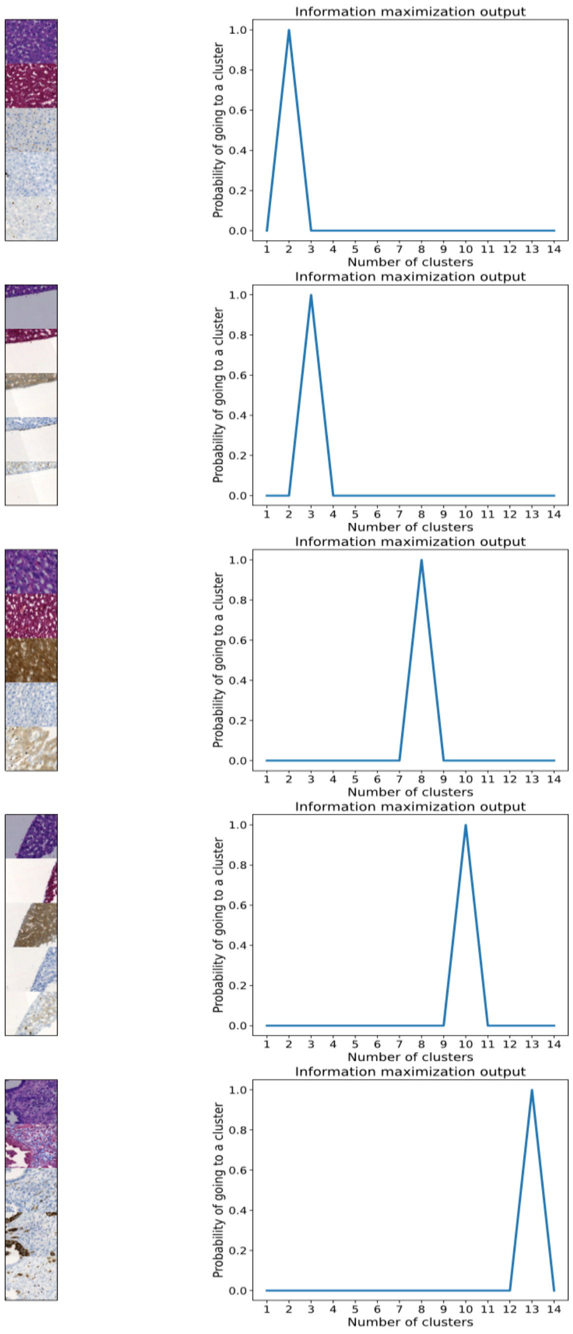

S5 Fig. Information maximization output of unused samples (128×128) during training using the 14-cluster set model

S5 Fig shows a few 128×128 pixels patches and their clustering outcome using the 14-cluster set model. We did not use these few samples for training the model. Even so, if we compare their clustering result with the training results of the 14-cluster set using 128×128 pixels patches, we will notice that they are very consistent.
